# Medical management of intraluminal inferior vena cava thrombus from hemostatic agent after extreme lateral interbody fusion

**DOI:** 10.1093/jscr/rjaf980

**Published:** 2025-12-12

**Authors:** Autumn Pak, Kevin Stehlik, Justin Smith, Drazen Petrinec

**Affiliations:** Department of General Surgery, Summa Health - Akron City Hospital, 55 Arch St, Suite 2F, Akron, OH 44304, United States; Department of Orthopedics, Summa Health - Akron City Hospital, 141 Forge St, Akron, OH 44304, United States; Department of General Surgery, Summa Health - Akron City Hospital, 55 Arch St, Suite 2F, Akron, OH 44304, United States; Department of Vascular Surgery, Summa Health - Akron City Hospital, 55 Arch St, Suite 215, Akron, OH 44304, United States

**Keywords:** hemostasis, coagulation cascade, thromboembolic event

## Abstract

There are a variety of hemostatic agents that can be used to control intraoperative bleeding, by creating a hemostatic and adhesive sealing effect. Despite their effectiveness, the use of such agents is not without risks. We present a case about the inadvertent injection of gelatin-based hemostatic agents into the inferior vena cava during spinal surgery, which was successfully managed without the need for vascular intervention. While the patient in this case experienced a favorable outcome with conservative management, this case underscores the importance of appropriate technique in the use of hemostatic agents.

## Introduction

Locally applied hemostatic agents are used in a variety of surgical procedures. Hemostatic agents can be composed of a variety of materials and work by triggering the coagulation cascade, creating a hemostatic and adhesive sealing effect [1]. Combined flowable gelatin and thrombin-based hemostats have been shown to be cost effective and provide successful hemostasis. While originally employed in vascular surgery, these agents have found broader applications in various surgical specialties [2–4]. Surgiflo is a gelatin-based hemostatic agent containing thrombin that creates a matrix for platelet adherence and forming a platelet plug, thus aiding in hemostasis. Gelfoam is also gelatin-based but its absorbability allows it to absorb up to 45 times its weight, promoting tamponade and providing a stable matrix to promote clot formation [4]. Despite their effectiveness, the use of such agents is not without risks. Rare but significant complications have been documented in the literature. Consent has been obtained for this case report.

## Case report

**Figure 1:**
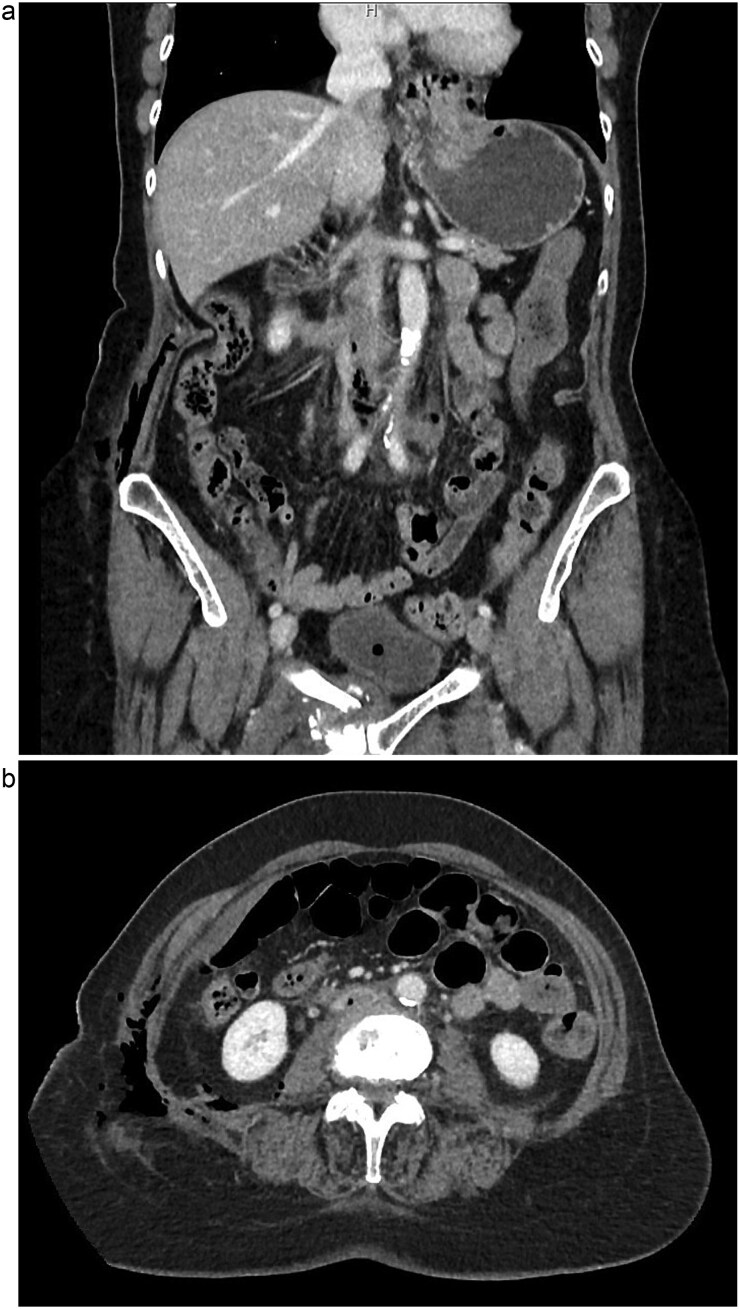
(a) Coronal view of thrombus and air in IVC, initial imaging. (b) Axial view of thrombus and air in IVC, initial imaging.

**Figure 2:**
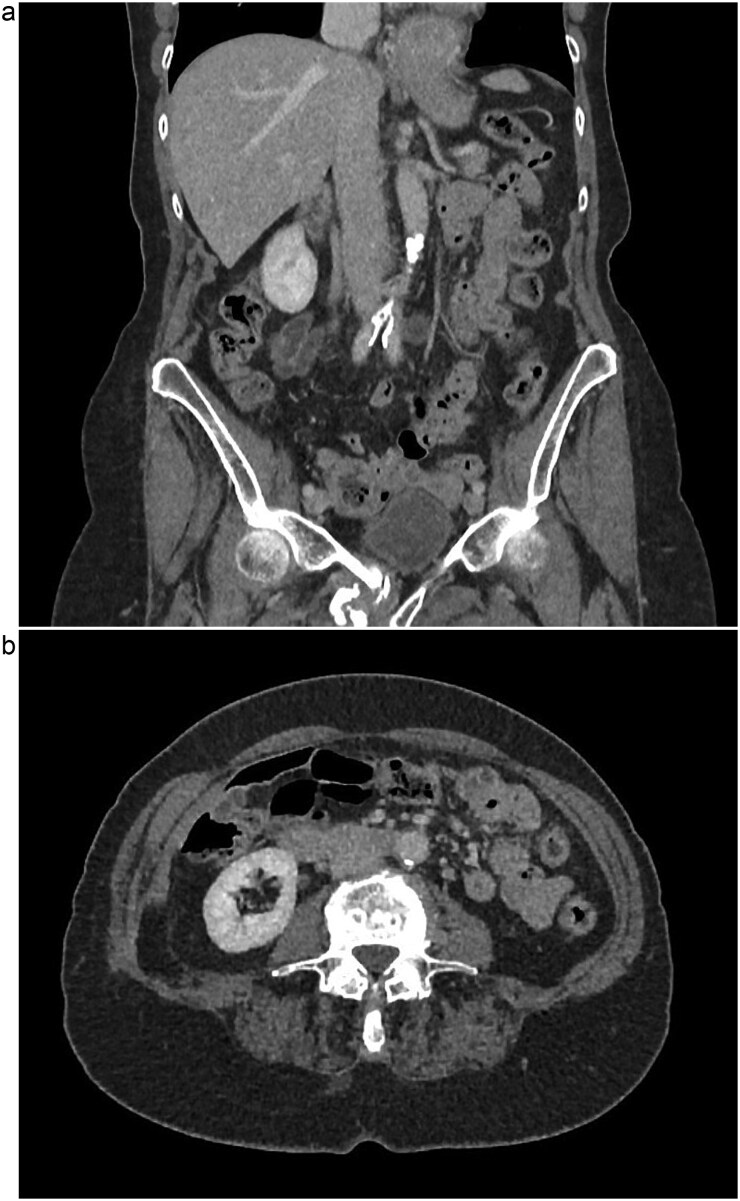
(a) Coronal view of resolved thrombus, 5 months post-op. (b) Axial view of resolved thrombus, 5 months post-op.

The patient is a 75-year-old female with no relevant past medical or surgical history who underwent an L4-L5 extreme lateral interbody fusion (XLIF) at an outside surgical center. During the procedure, a significant amount of blood loss from an undetermined bleeding vessel was encountered. The primary surgeon injected Surgiflo and Gelfoam to attempt to obtain hemostasis. The XLIF was completed with an estimated blood loss of greater than 2 l. In the post-anesthesia care unit (PACU), the patient remained hypotensive. Two units of packed red blood cells, phenylephrine, 5 l of crystalloid, and 50 g of albumin were administered in the PACU and the patient was transferred to a surgical intensive care unit at a level one trauma center. A computed tomography (CT) scan was obtained which demonstrated a thrombus present in the inferior vena cava (IVC) extending to the left common iliac vein (Fig. 1a and b). She received an additional unit of packed red blood cells and blood pressure support was maintained with norepinephrine. Vascular surgery was consulted. At the time of evaluation, the patient had remarkably improved hemodynamics and was on minimal pressor support. After discussion of risks and benefits of intervention versus medical management, the vascular team recommended therapeutic anticoagulation with continuous intravenous heparin. The patient was observed in the intensive care unit for 2 days until she was off pressor support. She was started on apixaban once her hemoglobin was stable on three consecutive blood draws. A repeat CT angiogram 1 month after discharge demonstrated near resolution with a small residual thrombus in the left common iliac vein (Fig. 2a and b). She has returned to normal activity and will remain on apixaban for 6 months after the inciting event and the thrombus will be evaluated again for further evolution.

## Discussion

Thrombin-based hemostatic agents are widely utilized across surgical specialties to control bleeding, particularly in challenging or minimally accessible operative fields. While their efficacy is well-established, rare but serious complications such as thromboembolism can occur, especially when used in proximity to major vascular structures [5–8].

Surgiflo, a gelatin-thrombin matrix, and Gelfoam, an absorbable gelatin sponge, are commonly employed due to their ease of use and hemostatic reliability. However, when these agents inadvertently enter the venous system, they can induce thrombosis through the unregulated activation of the coagulation cascade [8]. In this patient, significant intraoperative blood loss led the surgical team to aggressively pursue hemostasis with these agents, unintentionally causing intraluminal exposure.

This complication has been reported but a handful of times in the literature. Other reported adverse outcomes associated with topical hemostatic agents include thromboembolic events, abscess formation, cauda equina syndrome, and hypersensitivity reactions [5–8]. Management strategies in such scenarios range from emergent surgical intervention to conservative medical management, often tailored to the patient’s stability and overall clinical status.

Our patient was hemodynamically unstable post-operatively but responded well to fluid resuscitation, vasopressors, and blood transfusion. Given her improving condition and the absence of embolic symptoms, the vascular surgery team elected to pursue medical management with therapeutic anticoagulation rather than immediate surgical or endovascular intervention. This approach led to near-complete resolution of the thrombus on follow-up imaging and avoided the risks associated with operative thrombectomy.

This case reinforces the importance of careful use of thrombin-containing agents in surgeries near large vessels. In such cases, judicious application, awareness of the surrounding anatomy, and appropriate patient monitoring post-operatively are essential. Moreover, early recognition and multidisciplinary management of complications can lead to favorable outcomes without necessitating invasive interventions.

## Conclusion

Thrombus formation following the use of thrombin-based hemostatic agents is an uncommon but serious complication, particularly in surgeries involving exposure near major vascular structures such as the IVC. In our case, the patient was successfully managed with anticoagulation alone, avoiding the need for surgical intervention. While these agents remain invaluable tools for achieving hemostasis, their application should be guided by anatomical considerations and awareness of potential systemic absorption. Prompt diagnosis, close post-operative monitoring, and individualized management plans are critical to optimizing outcomes in patients experiencing such complications.

## Presentation

This case report was presented at the NEOMED 36th Annual Regional Scholarship Day, Rootstown, OH, United States, 14 May 2025.
